# Asymmetric figure-of-eight single-layer suture technique for intestinal anastomosis: A preliminary study

**DOI:** 10.3389/fsurg.2023.1109751

**Published:** 2023-02-13

**Authors:** Mingzhu Liu, Mingxiang Zhang, Xiang Ren, Chen Liu, Huaijing Yu, Xiao-Liang Xu, Guo-Jian Ding, Tingliang Fu, Lei Geng, Fengchun Cheng

**Affiliations:** ^1^ Department of Pediatric Surgery, Binzhou Medical University Hospital, Binzhou, China; ^2^ Department of General Surgery, Boxing People's Hospital, Boxing, China; ^3^ Department of Anorectal Surgery, Binzhou Medical University Hospital, Binzhou, China; ^4^ Department of Surgery, Shanghai Children's Hospital, Shanghai Jiao Tong University, Shanghai, China; ^5^ Department of Pediatric Surgery, Qilu Hospital of Shandong University Dezhou Hospital, Dezhou, China

**Keywords:** intestinal anastomosis, asymmetric figure-of-eight suture, postoperatively complications, pediatric, single layer suture

## Abstract

**Background:**

Anastomotic leakage is a life-threatening complication. Improvement of the anastomosis technique is needed, especially in patients with an inflamed edematous intestine. The aim of our study was to evaluate the safety and efficacy of an asymmetric figure-of-eight single-layer suture technique for intestinal anastomosis in pediatric patients.

**Methods:**

A total of 23 patients underwent intestinal anastomosis at the Department of Pediatric Surgery of Binzhou Medical University Hospital. Demographic characteristics, laboratory parameters, anastomosis time, duration of nasogastric tube placement, day of first postoperative bowel movement, complications, and length of hospital stay were statistically analyzed. The follow-up was conducted for 3–6 months after discharge.

**Results:**

Patients were divided into two groups: the single-layer asymmetric figure-of-eight suture technique (group 1) and the traditional suture technique (group 2). Body mass index in group 1 was lower than in group 2 (14.43 ± 3.23 vs. 19.38 ± 6.74; *P* = 0.036). The mean intestine anastomosis time in group 1 (18.83 ± 0.83 min) was less than that in group 2 (22.70 ± 4.11 min; *P* = 0.005). Patients in group 1 had an earlier first postoperative bowel movement (2.17 ± 0.72 vs. 2.80 ± 0.42; *P* = 0.023). The duration of nasogastric tube placement in group 1 was shorter than that in group 2 (4.12 ± 1.42 vs. 5.60 ± 1.57; *P* = 0.043). There was no significant difference in laboratory variables, complication occurrence, and length of hospital stay between the two groups.

**Conclusion:**

The asymmetric figure-of-eight single-layer suture technique for intestinal anastomosis was feasible and effective. More studies are needed to compare the novel technique with the traditional single-layer suture.

## Introduction

Intestinal anastomosis is a basic technique to restore gut continuity. Several intestinal anastomosis techniques, including hand-sewn, stapled, laparoscopic, robotic, and sutureless anastomoses, are used with varied outcomes (1–8). The hand-sewn intestinal anastomosis technique is divided into single-layer and double-layer suture techniques (9–12). Interrupted single-layer anastomosis is the gold standard owing to the occurrence of fewer complications (13). However, the smaller diameter of the intestinal wall in the pediatric population and a pathological status, including the presence of edema, ischemia, and inflammation, may make implementing the single-layer suture technique difficult. Moreover, the higher occurrence of postoperative complications may encourage surgeons to change their strategy to temporary intestinal stoma and two-stage procedures, among others (12–15). Thus, some patients may have to face several possible complications arising from multiple procedures. Herein, we modified an asymmetric figure-of-eight single-layer suture technique on the basis of an abdominal wall closure technique described by Höllwarth (16) and an *in vitro* porcine experimental finding (17). The asymmetric figure-of-eight single-layer suture technique has some advantages; it is easy to perform, is time-saving, and has better mucosal apposition (17). We piloted the modified suture technique by performing intestinal anastomoses, especially in the bowels with a pathological status in pediatric patients. We also assessed its feasibility and efficacy.

## Methods

### Data collection

Between January 2020 and October 2022, 23 pediatric patients underwent intestinal anastomoses at the Department of Pediatric Surgery, Binzhou Medical University Hospital. The inclusion criteria were as follows: (1) age <18 years; (2) undergoing emergent or elective intestine anastomosis; (3) normal renal and liver function; and (4) no contraindications for intestinal anastomosis, such as being hemodynamically unstable, having a severe intraperitoneal abscess, and having severe shock. The exclusion criteria included the following: (1) patients who underwent anastomosis in the stomach or distal part of the rectum; and (2) with any of the above contraindications.

Variables included age, sex, body mass index (BMI), laboratory data (white blood cell count and hemoglobin, albumin, and C-reactive protein levels), length of postoperative hospital stay, time of anastomosis, duration of nasogastric tube placement, time of first postoperative bowel movement, and postoperative complications, including anastomotic bleeding, leakage, stricture, intra-abdominal abscess, pelvic collection, and abdominal distension. The time taken for the anastomosis (in minutes) was recorded from the beginning of the first stitch placement to the end of cutting extra suture material from the last stitch.

An anastomotic leak was defined as follows: (1) body temperature >38°C, persistent abdominal pain, and tachycardia; (2) signs of peritonitis or intra-abdominal abscess confirmed by abdominal ultrasound; (3) non-absorbable material draining from a wound after oral administration; (4) elevated leucocyte count; and (5) visible disruption of the suture line during re-exploration (18, 19).

### Procedures

**Figure 1:**
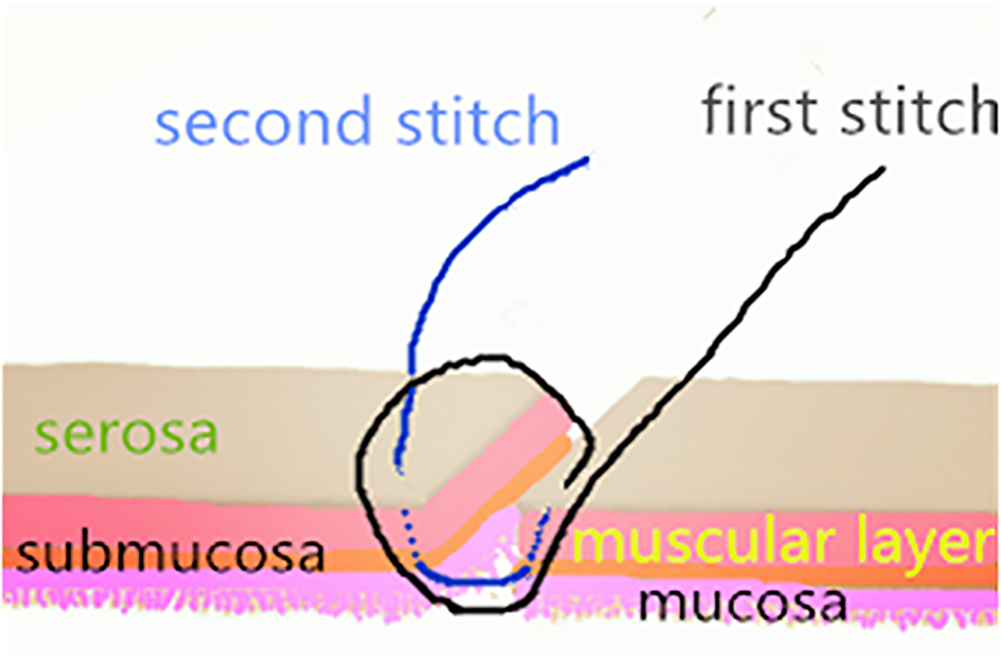
A schematic representation of the asymmetric figure-of-eight suture. The technique has been described in detail previously (17).

Patients were divided into two groups: the single-layer asymmetric figure-of-eight suture technique group (group 1) and the traditional suture technique, i.e., single-layer interrupted sutures, group (group 2) (9, 10, 20, 21). The single-layer asymmetric figure-of-eight suture technique has been described in detail by Liu et al. (17). To summarize, an absorbable surgical suture (4–0 or 5–0 Ethicon) was used for anastomosis. The first suture and withdrawal were conducted by taking a bite of 2 mm apart from the cut end and assured to include all layers of the intestine. The needle was obliquely inserted into the serosa, muscularis, and submucosa without mucosa to the contralateral layer. The second suture and withdrawal were obliquely inserted into the serosa, muscularis, and submucosa without mucosa to the contralateral submucosa, muscularis, and serosa by taking a bite 1 mm apart from the cut end and forward from the first suture, as shown in Figure 1. Before tying the knots, each suture should be tightly approximated to avoid cutting through the fragile bowel tissues.

All patients were subjected to the same postoperative program, including antibiotics, administration of fluid, and placement of a nasogastric tube.

The follow-up was conducted *via* telephone or outpatient department interview for 3–6 months.

### Ethical approval

All procedures performed in the present study were in accordance with the ethical standards of the 1964 Helsinki Declaration. Written informed consent was obtained from the parents and/or legal guardians of the patients.

### Statistical analysis

The differences between the groups were analyzed using the SPSS Statistics version 26.0 software (IBM Corp., Armonk, NY, USA). Continuous data were analyzed using the Student's *t*-test. Fisher's exact test and the Pearson chi-square test were used to analyze categorical data. A *P*-value <0.05 was considered statistically significant.

## Results

**Figure 2:**
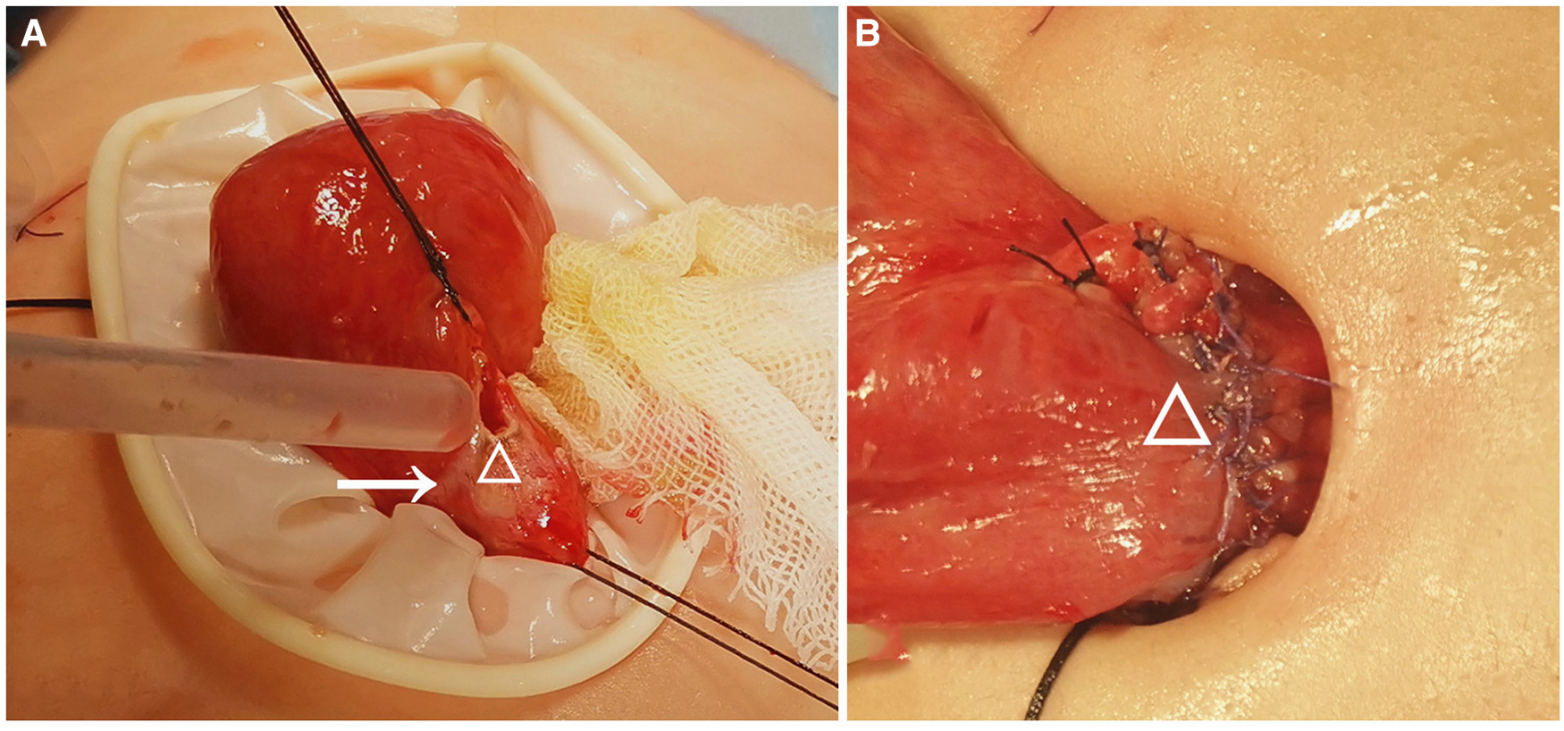
A 5-month-old boy with generalized peritonitis and septic shock caused by a perforated terminal ileum due to a congenital fibrotic band compression. (**A**) Perforation (small Δ) and necrosis (arrow) at the terminal ileum; (**B**) completion of primary anastomosis (large Δ) using the asymmetric figure-of-eight suture technique.

**Figure 3:**
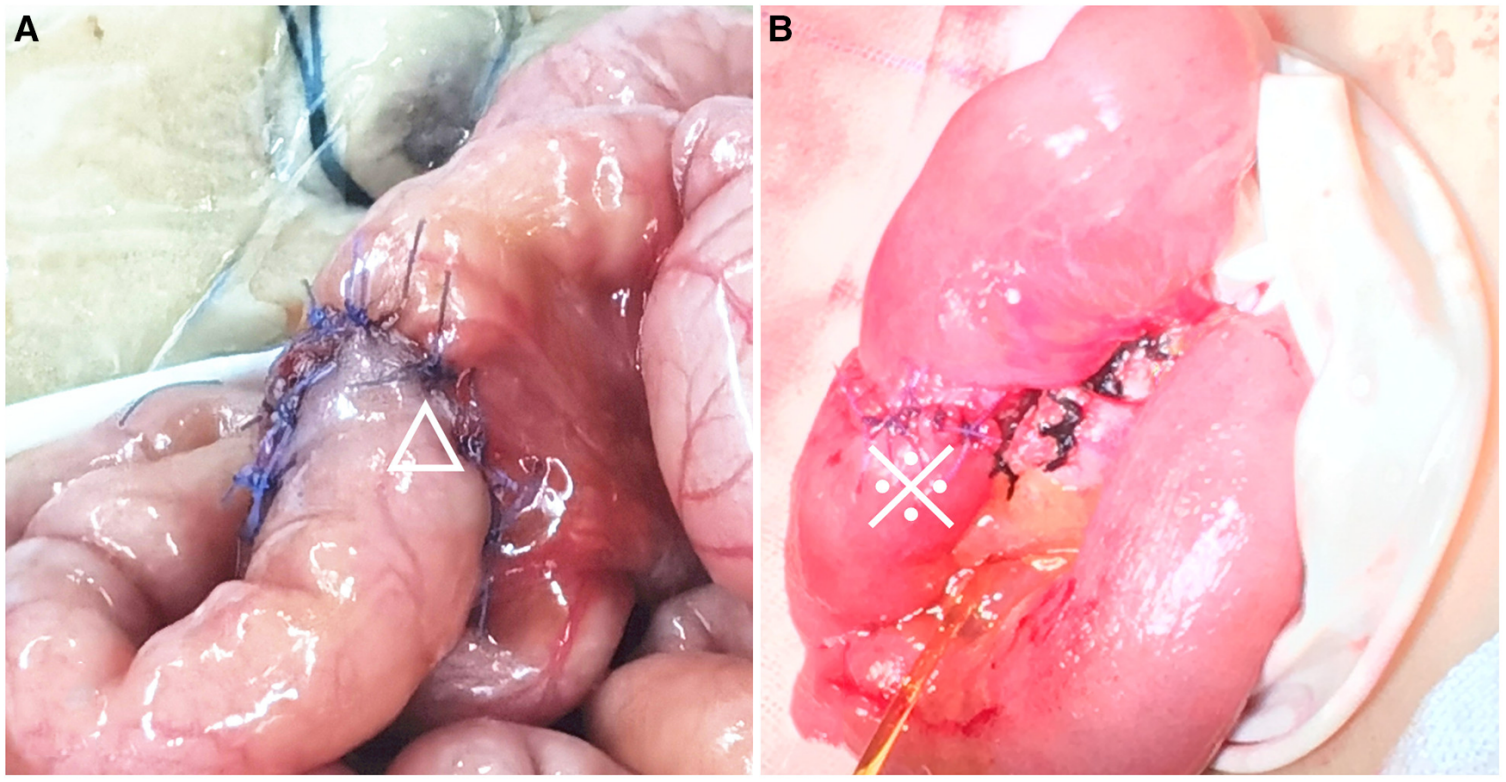
Comparison of the asymmetric figure-of-eight suture technique and traditional single-layer suture technique. (**A**) A 50-day-old boy with biliary atresia undergoing Kasai portoenterostomy, completion of end-to-side jejuno-jejunal anastomosis (△); (**B**) a six-year-old boy with Meckel's diverticulum complicated by strangulated internal hernia undergoing necrotic bowel resection and end-to-end ileoileal anastomosis (※).

A total of 23 pediatric patients who underwent anastomosis were assessed for eligibility, including 13 (56.52%) who underwent anastomosis with a single-layer asymmetric figure-of-eight suture technique (Figure 2) and 10 (43.48%) who underwent anastomosis with a traditional single-layer interrupted suture technique (Figure 3). Demographic and clinical characteristics are listed in Table 1. The male-to-female ratio was 2.29:1. The BMI was significantly lower in group 1 than in group 2 (14.43 ± 3.23 vs. 19.38 ± 6.74; *P* = 0.036). The mean intestine anastomosis time in group 1 (18.83 ± 0.83 min) was significantly lower than in group 2 (22.70 ± 4.11 min; *P* = 0.005). The patients in group 1 had an earlier first postoperative bowel movement than those in group 2 (2.17 ± 0.72 vs. 2.80 ± 0.42; *P* = 0.023). The duration of nasogastric tube placement in group 1 was significantly shorter than that in group 2 (4.12 ± 1.42 vs. 5.60 ± 1.57; *P* = 0.043). No significant differences were found between the two groups with regard to sex distribution (*P* = 0.405), median age (*P* = 0.065), serum albumin level (*P* = 0.084), serum hemoglobin level (*P* = 0.054), C-reactive protein (*P* = 0.636), and white blood cell count (*P* = 0.230). Demographic and clinical characteristics are listed in Table 1.

**Table 1.** Demographic and clinical characteristics.

	Group 1 (*n* = 13)	Group 2 (*n* = 10)	*P*-value
**Sex (*n*)**			0.405
Male	8	8	
Female	5	2	
Median age (IQR)	14 (1.25-311.25)	400.5 (15.25-1822.5)	0.065
BMI	14.43 ± 3.23	19.38 ± 6.74	0.036
**Laboratory findings**			
Albumin (g/L)	39.23 ± 3.96	39.49 ± 6.15	0.907
Hemoglobin (g/L)	153.00 ± 47.12	116.30 ± 34.15	0.054
WBC (×10^9^/L)	14.96 ± 6.42	11.91 ± 4.86	0.230
CRP (mg/L)	18.38 ± 52.98	9.81 ± 20.45	0.636
Anastomosis time (min)	18.83 ± 0.83	22.70 ± 4.11	0.005
Duration of nasogastric tube placement (days)	4.12 ± 1.42	5.60 ± 1.57	0.043
First postoperative bowel movement (days)	2.17 ± 0.72	2.80 ± 0.42	0.023
Length of hospital stay (days)	13.17 ± 5.35	15.00 ± 7.74	0.520

Values are given as mean ± standard deviation unless otherwise indicated.

IQR, interquartile range; BMI, body mass index; WBC, white blood cells; CRP, C-reactive protein.

Ileo-ileal, jejuno-jejunal, and portojejunal anastomoses were performed in groups 1 and 2. Major causes included gut malrotation, Meckel's diverticulum, perforation, gut or biliary atresia, duplication, small bowel obstruction, or neonatal necrotizing enterocolitis.

No major postoperative complications were found in any of the children.

## Discussion

Intestinal anastomosis is an essential surgical technique to restore intestinal continuity (1). In the pediatric population, the primary intestinal anastomosis technique is usually chosen according to the patient's general status and intestinal condition. The disease spectrum includes neonatal necrotizing enterocolitis, Meckel's diverticulum with complications, intestinal atresia, and strangulated bowel obstruction. A feasible and easy-to-perform intestinal anastomotic technique is necessary to reduce major postoperative complications, especially for those who have severely altered intestinal morphology. A safe and efficacious bowel anastomosis includes the following technical aspects: meticulous technique; gentle tissue handling; accurate mucosa apposition; and avoidance of tension and ischemia at the anastomotic site (1, 9, 19, 22).

Although the use of the traditional double-layer intestinal anastomosis or single-layer suture technique for intestinal anastomosis is controversial (23), the single-layer extramucosal suture anastomosis technique is widely accepted by pediatric surgeons (14). In the present study, we created an easy-to-perform anastomotic technique with two stitches at different horizontal levels (17), which might minimize the tension and prevent the surgeon from cutting through the intestinal wall while tying the knot, especially in inflamed edematous intestinal walls (17, 24).

Several studies have reported that the early removal of the nasogastric tube and initiation of oral feeding can promote the recovery of intestinal function, shorten the length of the hospital stay, and prevent intestinal obstruction (25–28). In the present study, the mean duration of nasogastric tube placement was significantly shorter in group 1 than in group 2. The first day of postoperative bowel movement in group 1 was significantly shorter than that in group 2, suggesting a more rapid intestinal function recovery.

Our results showed that the mean anastomosis time was significantly shortened in the asymmetric figure-of-eight single-layer suture technique group than in the single-layer interrupted sutures group, partly owing to the requirement of fewer knots (17). Time-saving is also important for patients who experience severe complications pre- or intraoperatively, such as intestinal volvulus and shock. Postoperative complications, such as abscess or anastomotic leakage, were absent even in patients with high-risk primary anastomosis, such as necrotizing enterocolitis, and generalized peritonitis.

## Limitations

This was a retrospective study. The sample size was relatively small, and the study was prone to the incomplete collection of individual data. Owing to the absence of a guideline to adhere to, the availability of intestinal anastomosis options may have been affected by the training or previous experience of pediatric surgeons. Furthermore, all patients were treated in a single medical center, and the results are not representative of other medical centers. Therefore, a multi-institutional trial with a large sample size determined using power analysis is required in the future.

## Conclusion

The asymmetric figure-of-eight single-layer suture technique was feasible and efficacious. It was superior to the traditional single-layer technique for intestinal anastomosis. Further studies are needed to confirm the preliminary findings.

## Data Availability

The raw data supporting the conclusions of this article will be made available by the authors, without undue reservation.

## References

[1] Chen C. The art of bowel anastomosis. Scand J Surg. (2012) 101(4):238–40. 10.1177/145749691210100403 23238497

[2] Madani R Day N Kumar L Tilney HS Gudgeon AM. Hand-sewn versus stapled closure of loop ileostomy: a meta-analysis. Dig Surg. (2019) 36(3):183–94. 10.1159/000487310 29514142

[3] Naoi D Horie H Koinuma K Kumagai Y Ota G Tojo M Intestinal mucosa staple line integrity and anastomotic leak pressure after healing in a porcine model. Surg Today. (2021) 51(10):1713–9. 10.1007/s00595-021-02267-9 33743053

[4] Sedano JVR Castro BA Alelu RM Vázquez AG Fraile AG Novillo IC. Use of 5-mm staple in neonatal intestinal surgery. J Laparoendosc Adv Surg Tech A. (2021) 31(9):1092–5. 10.1089/lap.2021.0181 34252323

[5] Uppal A Pigazzi A. New technologies to prevent anastomotic leak. Clin Colon Rectal Surg. (2021) 34(6):379–84. 10.1055/s-0041-1735268 34853558 PMC8610641

[6] Giaccaglia V Antonelli MS Franceschilli L Salvi PF Gaspari AL Sileri P. Different characteristics of circular staplers make the difference in anastomotic tensile strength. J Mech Behav Biomed Mater. (2016) 53:295–300. 10.1016/j.jmbbm.2015.08.029 26379251

[7] Saeidi H Opfermann JD Kam M Wei S Leonard S Hsieh MH Autonomous robotic laparoscopic surgery for intestinal anastomosis. Sci Robot. (2022) 7(62):eabj2908. 10.1126/scirobotics.abj2908 35080901 PMC8992572

[8] Fan C Ma J Zhang HK Gao R Li JH Yu L Sutureless intestinal anastomosis with a novel device of magnetic compression anastomosis. Chin Med Sci J. (2011) 26(3):182–9. 10.1016/s1001-9294(11)60046-1 22207929

[9] Kar S Mohapatra V Singh S Rath PK Behera TR. Single layered versus double layered intestinal anastomosis: a randomized controlled trial. J Clin Diagn Res. (2017) 11(6):Pc01–pc4. 10.7860/jcdr/2017/24817.9983 28764239 PMC5535431

[10] Singh R Najmi HI Chahal RK Nikhil D. A comparative study of single-layered versus double-layered intestinal anastomosis. Cureus. (2022) 14(3):e23088. 10.7759/cureus.23088 35464566 PMC8996431

[11] Sajid MS Siddiqui MR Baig MK. Single layer versus double layer suture anastomosis of the gastrointestinal tract. Cochrane Database Syst Rev. (2012) 1:Cd005477. 10.1002/14651858.CD005477.pub4 22258964 PMC12107684

[12] Close K Epstein KL Sherlock CE. A retrospective study comparing the outcome of horses undergoing small intestinal resection and anastomosis with a single layer (Lembert) or double layer (simple continuous and cushing) technique. Vet Surg. (2014) 43(4):471–8. 10.1111/j.1532-950X.2014.12143.x 24689880

[13] Leslie A Steele RJ. The interrupted serosubmucosal anastomosis-still the gold standard. Colorectal Dis. (2003) 5(4):362–6. 10.1046/j.1463-1318.2003.00460.x 12814417

[14] Ordorica-Flores RM Bracho-Blanchet E Nieto-Zermeño J Reyes-Retana R Tovilla-Mercado JM Leon-Villanueva V Intestinal anastomosis in children: a comparative study between two different techniques. J Pediatr Surg. (1998) 33(12):1757–9. 10.1016/s0022-3468(98)90279-2 9869045

[15] Nieto JE Dechant JE Snyder JR. Comparison of one-layer (continuous Lembert) versus two-layer (simple continuous/cushing) hand-sewn end-to-end anastomosis in equine jejunum. Vet Surg. (2006) 35(7):669–73. 10.1111/j.1532-950X.2006.00206.x 17026553

[16] Höllwarth ME. Short bowel syndrome. In: Puri P Höllwarth ME, editors. Pediatric surgery. Springer surgery atlas series. Berlin, Heidelberg: Springer (2006). p. 264–74.

[17] Liu C Wang Y Zhao AR Hu FA Fan Q Han G An alternative asymmetric figure-of-eight single-layer suture technique for bowel anastomosis in an in vitro porcine model. Front Surg. (2022) 9:896542. 10.3389/fsurg.2022.896542 36248362 PMC9554239

[18] Burch JM Franciose RJ Moore EE Biffl WL Offner PJ. Single-layer continuous versus two-layer interrupted intestinal anastomosis: a prospective randomized trial. Ann Surg. (2000) 231(6):832–7. 10.1097/00000658-200006000-00007 10816626 PMC1421072

[19] Aniruthan D Pranavi AR Sreenath GS Kate V. Efficacy of single layered intestinal anastomosis over double layered intestinal anastomosis-an open labelled, randomized controlled trial. Int J Surg. (2020) 78:173–8. 10.1016/j.ijsu.2020.04.066 32387214

[20] Bailey HR LaVoo JW Max E Smith KW Butts DR Hampton JM. Single-layer polypropylene colorectal anastomosis. Experience with 100 cases. Dis Colon Rectum. (1984) 27(1):19–23. 10.1007/bf02554066 6360594

[21] Slieker JC Daams F Mulder IM Jeekel J Lange JF. Systematic review of the technique of colorectal anastomosis. JAMA Surg. (2013) 148(2):190–201. 10.1001/2013.jamasurg.33 23426599

[22] Goulder F. Bowel anastomoses: the theory, the practice and the evidence base. World J Gastrointest Surg. (2012) 4(9):208–13. 10.4240/wjgs.v4.i9.208 23293735 PMC3536859

[23] Milone M Elmore U Allaix ME Bianchi PP Biondi A Boni L Fashioning enterotomy closure after totally laparoscopic ileocolic anastomosis for right colon cancer: a multicenter experience. Surg Endosc. (2020) 34(2):557–63. 10.1007/s00464-019-06796-w 31011862

[24] Awad S El-Rahman AIA Abbas A Althobaiti W Alfaran S Alghamdi S The assessment of perioperative risk factors of anastomotic leakage after intestinal surgeries; a prospective study. BMC Surg. (2021) 21(1):29. 10.1186/s12893-020-01044-8 33413244 PMC7789647

[25] Greer D Karunaratne YG Karpelowsky J Adams S. Early enteral feeding after pediatric abdominal surgery: a systematic review of the literature. J Pediatr Surg. (2020) 55(7):1180–7. 10.1016/j.jpedsurg.2019.08.055 31676081

[26] Peng Y Xiao D Xiao S Yang L Shi H He Q Early enteral feeding versus traditional feeding in neonatal congenital gastrointestinal malformation undergoing intestinal anastomosis: a randomized multicenter controlled trial of an enhanced recovery after surgery (ERAS) component. J Pediatr Surg. (2021) 56(9):1479–84. 10.1016/j.jpedsurg.2021.02.067 33838898

[27] Yadav PS Choudhury SR Grover JK Gupta A Chadha R Sigalet DL. Early feeding in pediatric patients following stoma closure in a resource limited environment. J Pediatr Surg. (2013) 48(5):977–82. 10.1016/j.jpedsurg.2013.02.013 23701770

[28] Tian Y Zhu H Gulack BC Alganabi M Ramjist J Sparks E Early enteral feeding after intestinal anastomosis in children: a systematic review and meta-analysis of randomized controlled trials. Pediatr Surg Int. (2021) 37(3):403–10. 10.1007/s00383-020-04830-w 33595685

